# Rapid, moderate, or slow bleeding? CT analysis of abdominopelvic active vascular contrast extravasation classes and mortality outcomes

**DOI:** 10.1007/s00330-025-11693-z

**Published:** 2025-05-21

**Authors:** Gun Chomchalerm, Rathachai Kaewlai, Sasima Tongsai, Jitti Chatpuwaphat, Somrach Thamtorawat, Banjerd Praditsuktavorn, Worapat Maitriwong, Anchisa Chatkaewpaisal, Pramuk Khamman, Junichi Matsumoto

**Affiliations:** 1 Department of Radiology, Faculty of Medicine Siriraj Hospital, Mahidol University, Bangkok, Thailand; 2 Department of Research, Faculty of Medicine Siriraj Hospital, Mahidol University, Bangkok, Thailand; 3 Department of Surgery, Faculty of Medicine Siriraj Hospital, Mahidol University, Bangkok, Thailand; 4 Department of Anatomy, Faculty of Medicine Siriraj Hospital, Mahidol University, Bangkok, Thailand; 5 Department of Emergency and Critical Care Medicine, St. Marianna University School of Medicine, Kawasaki, Japan

**Keywords:** Contrast extravasation, blood, Tomography, X-ray computed, Hemorrhage, Mortality, Adults

## Abstract

**Objectives:**

Building on prior findings that active vascular contrast extravasation (AVCE) size is an independent predictor of in-hospital mortality in abdominopelvic hemorrhages, this study aimed to categorize AVCEs using latent profile analysis (LPA) and examine differences in patient characteristics, treatments, and outcomes.

**Methods:**

We retrospectively included consecutive adults with CT-detected AVCE between January 2019 and May 2022. LPA was applied to classify AVCEs based on size-related features, optimizing the number of classes predictive of 24-h and in-hospital mortality. These classes were compared using univariable analysis with post-hoc pairwise comparisons to identify significant differences. Cutoff values for categorization were derived from size parameters and changes across arterial (AP) and portovenous (PVP) phases.

**Results:**

LPA classified 223 patients with single-organ, traumatic, and nontraumatic AVCEs (mean age 59.8 ± 20.1 years, 123 men) into three groups-slow (*n* = 136), moderate (*n* = 75), and rapid (*n* = 12). Slow AVCEs showed smaller size parameters and minimal changes between AP and delayed phases. Rapid AVCEs frequently exhibited coexisting pseudoaneurysms, smaller areas on AP, lower mean attenuation differences in AP-PVP pairs, and were associated with lower systolic and diastolic blood pressures, requiring the highest quantity of packed red cells. Perimeter percentage changes between AP and PVP performed comparably to LPA classes and provided practical classification cutoffs.

**Conclusion:**

LPA-based classification of AVCEs into slow, moderate, and rapid types revealed distinct size patterns and associated clinical outcomes, offering a robust framework for risk stratification and guiding management of abdominopelvic hemorrhages.

**Key Points:**

***Question*** *It is unclear if the size of active vascular contrast extravasation (AVCE) is predictive of mortality in patients with abdominopelvic hemorrhage.*

***Findings*** *AVCEs could be classified by latent profile analysis into three groups: slow, moderate, and rapid, based on size at multiphasic CT with distinct mortality risks.*

***Clinical relevance*** *Practical cutoff values of perimeter percentage changes of AVCE between arterial- and portovenous-phase CT were identified for AVCE classification, potentially guiding clinical prioritization and management of patients with abdominopelvic hemorrhage.*

**Graphical Abstract:**

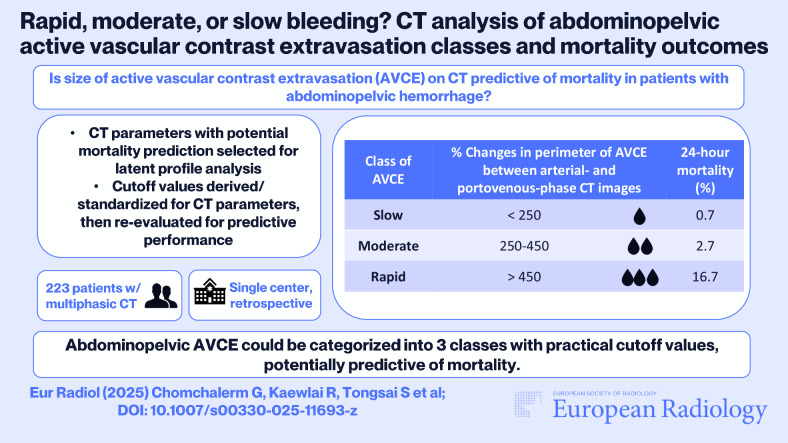

## Introduction

Abdominopelvic active vascular contrast extravasation (AVCE), a relatively infrequent yet serious condition known for its high morbidity and mortality, is typically diagnosed using computed tomography (CT) with intravenous contrast [1]. CT not only reveals the source of internal bleeding but also indicates its activity—whether it is ongoing or has stopped. This phenomenon was first observed in a patient with blunt traumatic splenic rupture in 1989 [2], with subsequent research consistently attesting to CT’s reliability in diagnosing and localizing AVCE [3–6]. The presence of AVCE often signals immediate intervention to control bleeding, typically involving medications, blood transfusion, endoscopy, transarterial embolization (TAE), surgery, or a combination thereof [3, 5–7].

The choice of an optimal bleeding control technique depends significantly on the hemorrhage’s location. Different studies have reported varying success rates of bleeding control based on the specific site of the hemorrhage. Some advocate for immediate TAE or surgery when AVCE is detected [5, 7–9], while others have demonstrated the success of conservative management in certain AVCEs [10–13]. For example, Lukies et al found that conservative management of AVCE associated with nontraumatic retroperitoneal hemorrhage was more effective than TAE [11]. It seems plausible that AVCE in different anatomical locations has distinct natural histories and treatment options, while some common characteristics exist, such as risk factors, CT appearances, treatments, and outcomes. Building on prior findings that AVCE size independently predicts in-hospital mortality [14], we aimed to classify abdominopelvic AVCEs based on size-related features to assess their impact on mortality. We also examined associated variations in patient characteristics, treatments, and outcomes.

## Materials and methods

### Study design and patient selection

**Fig. 1:**
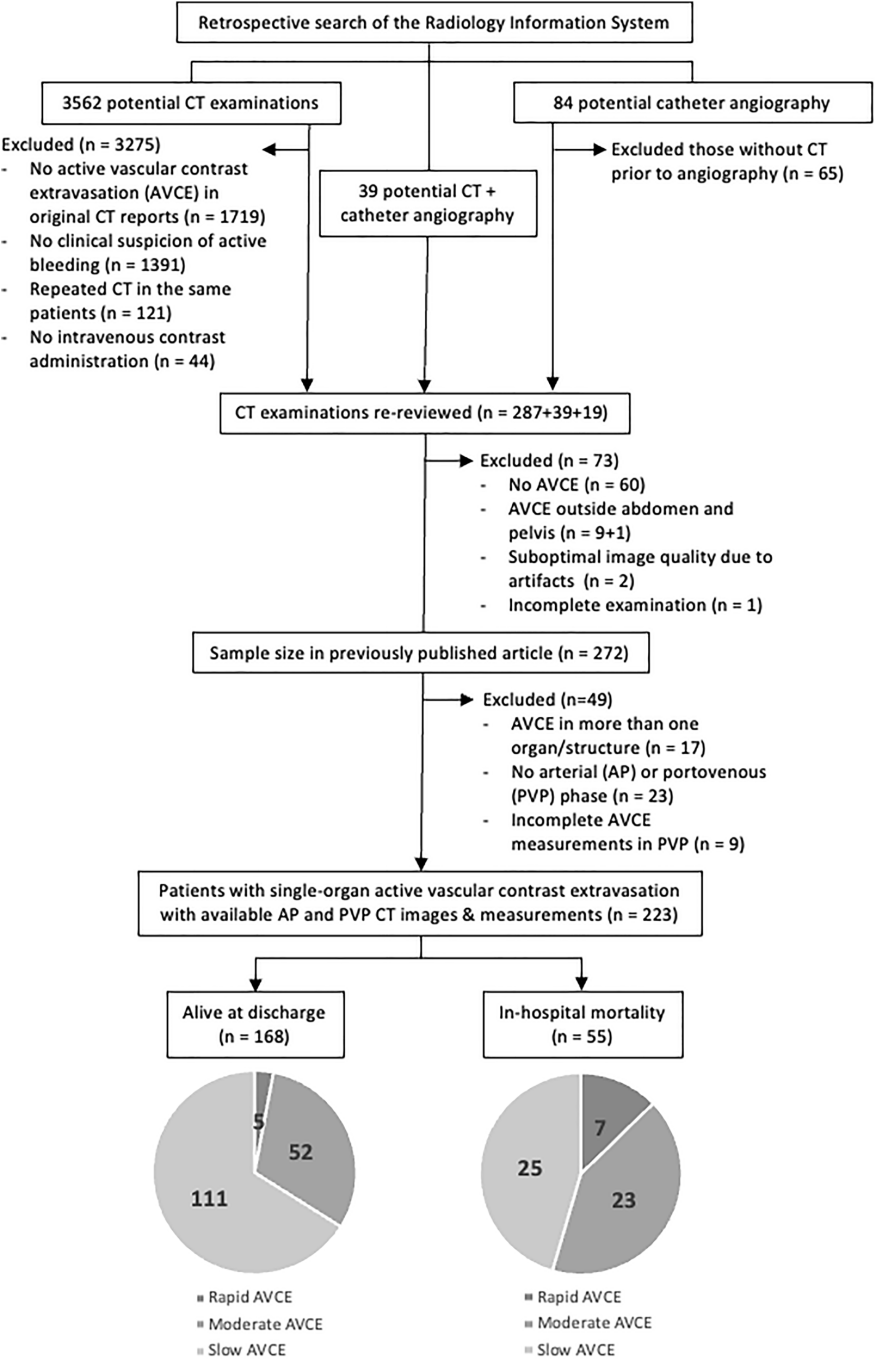
Flowchart of patient inclusion

The retrospective single-center study was carried out at a 2200-bed, trauma-level-1-equivalent academic hospital and approved by the Institutional Review Board (certificate of approval number Si 555/2022). The informed consent was waived due to its retrospective nature. Figure 1 shows a patient inclusion flowchart. The electronic database of the hospital’s Radiology Information System was searched between January 2019 and May 2022 for CT and catheter angiography of patients aged ≥ 18 years containing keywords related to active bleeding (i.e., “active contrast extravasation,” “active bleeding/hemorrhage,” “active bleeder”). Of 3685 CT and angiography reports identified, 3275 CTs and 65 angiographic studies were excluded, and 345 CT examinations remained for a re-review by an emergency radiologist (R.K.) with 20 years of experience for evidence of AVCE using standard definitions [1, 7]. Patients without AVCE (*n* = 60), having AVCE outside the abdomen and pelvis (*n* = 10), AVCE in more than one organ/structure (*n* = 17), lack of arterial (AP) or portovenous phase (PVP) CT (*n* = 32), suboptimal image quality and incomplete examination (*n* = 3) were further excluded from the investigation. A total of 223 CT examinations with AVCE were included in the final sample. This patient set had been evaluated in another previously published study [14] with different objective and endpoint.

### Clinical data abstraction

Two physicians (A.C. and P.K.) collected clinical information from electronic medical records for all eligible patients. Both possessed knowledge regarding the patients’ suspected history of active bleeding and the research objectives, but they remained blinded to specific locations of AVCE in each patient. The collected data included age, sex, initial physical examinations, laboratory data, coexistent diagnosis, durations, and treatment modalities. CT and angiographic findings were obtained from radiology reports by a radiologist (G.C.). To ensure accuracy and completeness, abstracted data underwent meticulous scrutiny by the corresponding author.

### Image acquisition

The CT examinations obtained at our institution (*n* = 203) used one of four multidetector CT scanners (Revolution CT, GE Healthcare), and axial images were reconstructed using a 1.25-mm slice thickness. The scan parameters were set to 120 kVp, 250 mAs, and a pitch ratio of 0.99, and at least one post-contrast phase (arterial phase; AP, portovenous phase; PVP, delayed phase; DP) was performed after intravenous contrast administration, covering the upper border of hemidiaphragms to ischial tuberosities. The AP, PVP, and DP were performed at 35–45 s, 70–80 s, and 5–10 min or more after contrast administration, respectively. For CT examinations performed at outside hospitals (*n* = 18), they had a maximum slice thickness of 2 mm, and at least one post-contrast phase was available. All images were in our Picture Archiving and Communication System (Synapse, Fujifilm Corporation). In total, the pre-contrast phase, AP, PVP and DP were performed in 221, 209, 219, and 158 patients, respectively.

### Image reinterpretation

All 223 CT scans were independently re-reviewed for AVCE spaces by two emergency radiologists (J.C. and R.K.) with 7 and 20 years of experience who were blinded to patient history details, clinical data, and diagnosis, except for patient age, sex, and clinical presentation suspicious of active bleeding. Each reviewer assigned a space for each AVCE based on perceived ease of spread (Supplementary Table E1), and all disagreements were resolved by a third radiologist (J.M.) who specialized in emergency imaging and intervention with 22 years of experience. Reports of angiography were reviewed for details, and any results inconsistent with CT findings were re-reviewed by an interventional radiologist (S.T.).

### Counts, CT characteristics of AVCE

The “representative” AVCE was the only one that a patient had in the CT examination. If there were more than one AVCE in the same organ or structure, the largest one was selected as a representative of the examination. All measurements were made on a standard workstation with an electronic caliper for obtaining the size and CT attenuation of AVCE. These values were recorded in terms of area (mm^2^), perimeter (mm), minimum diameter (mm), maximum diameter (mm), mean attenuation (Hounsfield units; HU), and standard deviation (SD) of attenuation (HU). They were calculated as percentages of changes between two pairs of CT phases (AP-PVP, AP-DP, and PVP-DP) as shown in the example: “(PVP-AP)/AP × 100.”

### Statistical analysis

Latent profile analysis (LPA) is an individual-focused statistical technique that identifies unobserved (latent) groups within a population by analyzing observed variable patterns. LPA assigns individuals probabilistically to latent profiles, each defined by unique variable combinations. This approach enables a more detailed understanding of population diversity, identifying groups with similar characteristics or circumstances [15]. In this study, LPA was applied to identify and classify distinct groups of patients based on CT parameters of AVCEs, enabling the characterization of latent patterns within the data to predict 24-h and in-hospital mortality. The analytical process in this study was as follows:

1. **Selection of CT parameters for mortality prediction**. CT parameters with potential prognostic value for mortality outcomes [14] were selected for LPA. These included size parameters (area, perimeter, minimum length, and maximum length), attenuation (mean density and standard deviation of density), and changes of these parameters between the AP and PVP phases.
2. **LPA using different combinations of CT parameters**. Six different methods of combining CT parameters (Table 1) were evaluated using LPA for their predictive ability regarding 24-h and in-hospital mortality. Among these, Method #6—comprising changes in area, perimeter, minimum length and maximum length between the AP and PVP phases—demonstrated the strongest predictive performance.
3. **Determination of the optimal number of LPA classes and their nomenclature**. Further evaluation of Method #6 was conducted to determine the most appropriate number of latent classes, ranging from 2 to 5 (Supplementary Table E2). Several statistical criteria, including the Bayesian Information Criterion (BIC), Integrated Complete-data Likelihood (ICL), and the Bootstrap Likelihood Ratio Test (BLRT), were utilized to identify the best-fitting model. The three-class model was selected as it provided a well-balanced and clinically meaningful categorization of AVCEs compared to the four-class model. These classes were designated as Slow, Moderate, and Rapid AVCEs. To identify unique and differentiating characteristics of each AVCE class (Supplementary Table E3), Pearson’s chi-square test and one-way ANOVA were used to examine potential factors associated with the three LPA-derived AVCE classes. Post-hoc Bonferroni correction or the Games-Howell test was applied for pairwise comparisons when appropriate. A unique characteristic of a class was defined as a feature that significantly distinguished it from both other classes, whereas a differentiating characteristic referred to a feature that significantly differed between two specific classes.
4. **Identifying the proxy for LPA-derived classes of AVCE**. Since the three-class LPA model relied on four CT parameters, applying all four in clinical practice would be impractical. To develop a more feasible approach while maintaining classification accuracy:
  - Cutoff values for individual patient CT parameters were established by first generating percentile distributions for each parameter across three distinct classes identified through LPA. These percentiles were then converted to a uniform scale, normalized, and categorized into groups for each of the four parameters. The standardized cutoff values are summarized in Table 2, with additional details provided in Supplementary File 1.
  - Forward stepwise multivariable logistic regression analysis was performed by reapplying the LPA-based classification to individual patient data using the established cutoff values.

**Table 1:** Latent profile analyses demonstrating different methods of combining computed tomographic features of active vascular contrast extravasation (AVCE) and the optimal number of classes that would be predictive of death at 24 h after admission and during admission

	Optimal number of AVCE class*	CT features	*p*-values of tested outcome data	
			Death in 24 h	In-hospital death
Method #1 (*n* = 209)	4	Area in PVP Perimeter in PVP Minimum length in PVP Maximum length in PVP Changes in … between AP-PVP pair Area Perimeter Minimum length Maximum length	0.004	0.046
Method #2 (*n* = 203)	3	Changes in … between AP-PVP pair Area Mean density SD of density	0.005	0.034
Method #3 (*n* = 203)	3	Changes in … between AP-PVP pair Minimum length Mean density SD of density	0.120	0.047
Method #4 (*n* = 202)	3	Changes in … between AP-PVP pair Perimeter Mean density SD of density	< 0.001	0.025
Method #5 (*n* = 202)	3	Changes in … between AP-PVP pair Maximum length Mean density SD of density	0.045	0.364
Method #6^+^ (*n* = 223)	3	Changes in … between AP-PVP pair Area Perimeter Minimum length Maximum length	0.003	0.004

*p*-values of < 0.05 are marked with an underline

The calculated samples in the table varied because certain parameters were unobtainable. For analysis, values must be available for all cases to be included

*AP* arterial phase, *PVP* portovenous phase

* Optimal number of classes of AVCE was determined by fit indices and Bootstrap Likelihood Ratio Test. The chosen method (#6)^+^ was eventually selected based on the superior ability to predict subsequent deaths. The optimal number of AVCE classes was 3 for practicality, which demonstrated similar predictive performance to 5 classes

**Table 2:** Cutoff values for the changes in size parameters of active vascular contrast extravasation between the arterial and portovenous phases of abdominopelvic CT

	Classes of AVCE		
Changes in size parameters between AP and PVP (%)	Slow	Moderate	Rapid
Area	< 800	800–1500	> 1500
Perimeter*	< 250	250–450	> 450
Minimum length	< 160	160–300	> 300
Maximum length	< 20	20–100	> 100

*AP* arterial phase, *AVCE* active vascular contrast extravasation, *CT* computed tomography, *PVP* portovenous phase

* Considered the best surrogate to represent LPA classes according to re-evaluation of variables based on forward stepwise binary logistic regression models

Categorical attributes such as sex, underlying diseases, medications, clinical history, laboratory results, and number of organs with AVCE were expressed using numbers and percentages. The mean and standard deviation (SD) were used to present continuous data, such as age, hospitalization duration, time until CT scan, and CT measurements, for normally distributed data. For non-normally distributed data, the median and range were presented. Statistical analyses were conducted using the MASS and mclust packages in R, as well as IBM SPSS Statistics for Windows, Version 26.0, with a significance threshold set at 0.05.

## Results

**Fig. 2:**
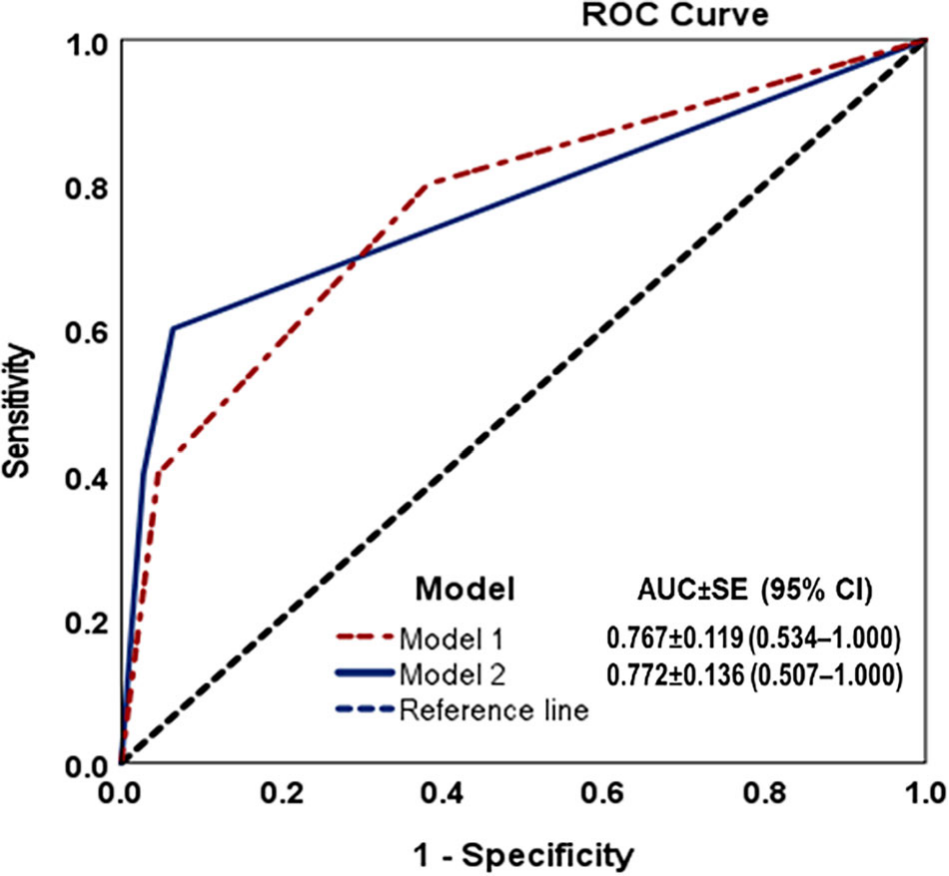
Comparison of ROC curves of Model 1 (utilizing CT classes based on latent profile analysis) and Model 2 (utilizing changes in perimeter between arterial and portovenous phases) in predicting deaths within 24 h of admission in patients with abdominopelvic active vascular contrast extravasation

There was a total of 223 representative AVCEs for the analysis, in which LPA was categorized as 136 in the slow class, 75 in the moderate class, and 12 in the rapid class. Table 3/Supplementary File 2 provides patient information, other CT findings, and treatment details, while Table 4/Supplementary File 2 demonstrates significant differences in changes in size parameters among these three classes. Multiple factors showed significant differences among the three AVCE classes in a univariable analysis, and Supplementary Table E3 summarizes unique characters of classes and differentiating characters between a pair of classes. Table 2 demonstrates cutoff values for the changes in size parameters between AP and PVP phases, in which the change in perimeter between AP and PVP was considered the proxy of LPA class (Fig. 2) (Table 5 and Supplementary File 1).

**Table 3:** Comparison of patient, treatment, and outcome characteristics among three computed tomographic classes of active vascular contrast extravasation (AVCE) of the abdomen and pelvis (*n* = 223)

	All (*n* = 223)	Slow AVCE (*n* = 136)	Moderate AVCE (*n* = 75)	Rapid AVCE (*n* = 12)	*p*-values
Mean age (years; SD)	59.8 (20.1)	58.3 (19.3)	61.5 (21.6)	65.2 (17.8)	0.344
Male sex (*n*, %)	123 (55.2)	78 (57.4)	41 (54.7)	4 (33.3)	0.275
Nontraumatic etiologies	169 (75.8)	99 (72.8)	60 (80.0)	10 (83.3)	0.414
Vital signs (mean, SD)					
Systolic blood pressure (mmHg)	123.3 (23.3)	123.8 (24.1)	125.8 (20.7)	**102.9** (**20.8)**	0.006
Diastolic blood pressure (mmHg)	72.6 (16.1)	73.3 (15.7)	73.6 (15.6)	**57.3** (**17.2)**	0.003
Pulse pressure (mmHg)	50.8 (18.9)	50.5 (19.7)	52.2 (18.4)	45.6 (11.5)	0.513
Pulse rate (beats/min)	95.8 (21.6)	95.4 (21.6)	97.2 (21.5)	92.3 (22.7)	0.703
Packed red cells					
Use (*n*, %)	183 (82.1)	106 (77.9)	67 (89.3)	10 (83.3)	0.118
Units in 24 h (median, min-max)	3 (0, 21)	3 (0, 17)	3 (0, 17)	**6** (**0, 21)**	0.005
Time intervals (h; median, min-max)					
From CT to angiography	5.4 (0.4, 148.3)	6.2 (0.4, 123.7)	4.8 (0.4, 148.3)	2.7 (1.1, 8.4)	0.096
From CT to surgery or other procedures	16.1 (0.4, 312.6)	18.4 (0.9, 201.7)	8.4 (0.4, 312.6)	3.5 (2.4, 17.5)	0.207
From CT to any treatment	5.3 (0.4, 201.7)	*6.1* (*0.4, 201.7)*	4.9 (0.4, 165.5)	*2.8* (*1.1, 8.4)*	0.046
Treatment combination					0.603
Angioembolization alone	56 (25.1)	39 (28.7)	17 (22.7)	0	
Angioembolization with endoscopy or surgery	67 (30.0)	35 (25.7)	27 (36.0)	5 (41.7)	
Endoscopy or surgery without angioembolization	60 (26.9)	39 (28.7)	17 (22.7)	4 (33.3)	
None of the above treatments	40 (17.9)	23 (16.9)	14 (18.7)	3 (25.0)	
ICU admission (*n*, %)	129 (57.8)	74 (54.4)	48 (64.0)	7 (58.3)	0.402
Length of stay (days; median, min-max)	17 (0, 355)	15.5 (0, 355)	18 (1, 179)	32 (1, 129)	0.142
Follow-up CT (*n*, %)	104 (46.6)	64 (47.1)	34 (45.3)	6 (50.0)	0.944
Rebleeding (*n*, %)	24 (10.9)	15 (11.2)	7 (9.3)	2 (16.7)	0.736
Discharge status (*n*, %)					0.011*
Improved	140 (62.8)	90 (66.2)	45 (60.0)	5 (41.7)	
Dead	55 (24.7)	*25* (*18.4)*	23 (30.7)	*7* (*58.3)*	
Transferred	28 (12.6)	21 (15.4)	7 (9.3)	0	
Death (*n*, %)					
Within 24 h of index CT	5 (2.2)	*1* (*0.7)*	2 (2.7)	*2* (*16.7)*	0.002
> 24 h to 7 days	13 (5.8)	7 (5.1)	4 (5.3)	2 (16.7)	0.257
> 7 days	37 (16.6)	17 (12.5)	17 (22.7)	3 (25.0)	0.119
Within the same admission (*n*, %)	55 (24.7)	*25* (*18.4)*	23 (30.7)	*7* (*58.3)*	0.003

*p*-values of < 0.05 are marked with an underline

The values in bold indicate values that exhibit a significant difference from the other two AVCE classes in a pairwise comparison

The values in italics indicate same-row pairs that exhibit a significant difference in a pairwise comparison

* The values for the discharge status ‘Dead’ were significantly different between the Slow AVCE and Rapid AVCE groups

**Table 4:** Comparison of computed tomography characteristics among three classes of active vascular contrast extravasation (AVCE) of the abdomen and pelvis (*n* = 223)

	All (*n* = 223)	Slow AVCE (*n* = 136)	Moderate AVCE (*n* = 75)	Rapid AVCE (*n* = 12)	*p*-value
Time from event to CT (h; median, min-max)	5.2 (0.1, 685.7)	4.9 (0.1, 685.7)	6.4 (1.0, 352.4)	6.8 (1.9, 30.1)	0.398
Site of AVCE* (*n*, %)					0.852
Gastrointestinal	51 (22.9)	26 (19.1)	22 (29.3)	3 (25.0)	
Peritoneum	30 (13.5)	20 (14.7)	9 (12.0)	1 (8.3)	
Mesentery	5 (2.2)	2 (1.5)	2 (2.7)	1 (8.3)	
Post-surgical spaces	6 (2.7)	4 (2.9)	2 (2.7)	0 (0)	
Extraperitoneum	11 (4.9)	5 (3.7)	5 (6.7)	1 (8.3)	
Retroperitoneum	40 (17.9)	23 (16.9)	14 (18.7)	3 (25.0)	
Spleen	2 (0.9)	2 (1.5)	0 (0)	0 (0)	
Subcutaneous tissues	7 (3.1)	6 (4.4)	1 (1.3)	0 (0)	
Adrenal glands	2 (0.9)	2 (1.5)	0 (0)	0 (0)	
Muscles	31 (13.9)	19 (14.0)	11 (14.7)	1 (8.3)	
Non-fat-containing tumors	1 (0.4)	0 (0)	1 (1.3)	0 (0)	
Kidneys	5 (2.2)	3 (2.2)	1 (1.3)	1 (8.3)	
Liver	14 (6.3)	9 (6.6)	4 (5.3)	1 (8.3)	
Ovary	1 (0.4)	1 (0.7)	0 (0)	0 (0)	
Pancreas	1 (0.4)	0 (0)	1 (1.3)	0 (0)	
Rectus sheath	15 (6.7)	13 (9.6)	2 (2.7)	0 (0)	
Uterus	1 (0.4)	1 (0.7)	0 (0)	0 (0)	
Space of AVCE* (*n*, %)					0.673
Free	92 (41.3)	52 (38.2)	35 (46.7)	5 (41.7)	
Loose	60 (26.9)	36 (26.5)	20 (26.7)	4 (33.3)	
Tight	71 (31.8)	48 (35.3)	20 (26.7)	3 (25.0)	
Coexistent pseudoaneurysm (*n*, %)	15 (6.7)	6 (4.4)	5 (6.7)	**4** (**33.3)**	< 0.001
Angiographically positive AVCE (*n*, %)	138 (61.9)	82 (60.3)	50 (66.7)	6 (50.0)	0.478
Surgically confirmed AVCE (*n*, %)	44 (19.9)	26 (19.3)	14 (18.7)	4 (36.4)	0.372
Area of AVCE (mm^2^; median, min-max)					
∆ between AP and PVP (%)	171.2 (−40.2, 3500)	**58.6** (**−40.2, 223.7)**	**300.8** (**27.6, 841.4)**	**1517.9** (**748.1, 3005)**	< 0.001
∆ between AP and DP (%)	313.8 (−77.4, 7825.7)	**183.0** (**−77.4, 2064.1)**	550.7 (−36.0, 3364.1)	4376.4 (929.5, 7825.7)	< 0.001
∆ between PVP and DP (%)	73.0 (−83.1, 1181.1)	72.1 (−83.1, 1181.1)	61.3 (−68.7, 459.7)	84.6 (−14.2, 278.3)	0.814
Perimeter of AVCE (mm; median, min-max)					
∆ between AP and PVP (%)	40.2 (−89.1, 770.6)	**17.0** (**−36.2, 91.8)**	**111.0** (**−89.1, 299.2)**	**477.1** (**253.0, 770.6)**	< 0.001
∆ between AP and DP (%)	93.1 (−70.5, 1353.1)	**57.3** (**−70.5, 395.0)**	175.4 (−34.4, 491.6)	836.0 (230.7, 1353.1)	< 0.001
∆ between PVP and DP (%)	28.5 (−68.4, 272.5)	29.2 (−68.4, 272.5)	27.5 (−63.4, 217.9)	50.6 (−40.0, 134.3)	0.866
Minimum length of AVCE (mm; median, min-max)					
∆ between AP and PVP (%)	43.9 (−48.6, 484.1)	**22.1** (**−48.6, 100.7)**	**113.5** (**−16.5, 416.8)**	**342.1** (**281.8, 484.1)**	< 0.001
∆ between AP and DP (%)	101.0 (−63.8, 1485.4)	**65.9** (**−63.8, 383.1)**	195.7 (−20.6, 662.9)	640.8 (267.0, 1485.4)	0.002
∆ between PVP and DP (%)	30.2 (−61.9, 378.2)	32.1 (−61.9, 378.2)	26.6 (−45.1, 265.7)	58.2 (−11.8, 315.3)	0.757
Maximum length of AVCE (mm; median, min-max)					
∆ between AP and PVP (%)	34.5 (−36.1, 786.4)	**19.7** (**−36.1, 108.2)**	**114.3** (**−18.8, 317.1)**	**440.8** (**157.8, 786.4)**	< 0.001
∆ between AP and DP (%)	88.5 (−60.3, 1288.0)	*59.5* (*−60.3, 427.2)*	174.5 (−35.8, 423.4)	*740.5* (*203.5, 1288.0)*	< 0.001
∆ between PVP and DP (%)	23.2 (−71.3, 316.8)	20.5 (−62.7, 316.8)	31.2 (−71.3, 186.6)	33.3 (−44.0, 163.8)	0.876
Mean attenuation of AVCE (HU; median, min-max)					
∆ between AP and PVP (%)	−7.5 (−80, 364.3)	−7.7 (−80, 280.9)	−2.4 (−50.9, 364.3)	**−21.8** (**−59.4, 12.8)**	0.011
∆ between AP and DP (%)	−28.1 (−77.7, 79.8)	−29.1 (−70.9, 45.4)	−26.7 (−73.1, 79.8)	−36.6 (−77.7, −4.7)	0.303
∆ between PVP and DP (%)	−21.7 (−63.9, 87.4)	−21.7 (−63.9, 87.4)	−23.0 (−62.8, 40.0)	−18.4 (−47.5, 14.4)	0.639
Standard deviation of attenuation of AVCE (HU; median, min-max)					
∆ between AP and PVP (%)	−7.9 (−77.3, 313.9)	*−11.9* (*−77.3, 196.0)*	*12.2* (*−51.9, 313.9)*	−24.6 (−43.8, −6.9)	0.001
∆ between AP and DP (%)	−31.8 (−76.8, 57.3)	*−39.7* (*−76.8, 57.3)*	*−16.7* (*−69.2, 47.1)*	NA	0.002
∆ between PVP and DP (%)	−30.7 (−74.2, 24.9)	−30.1 (−70.8, 24.9)	−25.9 (−74.2, 18.9)	−36.6 (−49.2, −4.9)	0.632

*p*-values of < 0.05 are marked with an underline

The values in bold indicate values that exhibit a significant difference from the other two AVCE classes in a pairwise comparison

The values in italics indicate same-row pairs that exhibit a significant difference in a pairwise comparison

*AP* arterial phase, *AVCE* active vascular contrast extravasation, *DP* delayed phase, *NA* not applicable, *PVP* portovenous phase, *HU* Hounsfield unit, *SD* standard deviation

* No. 1–4 = free space; No. 5–8 = loose space; No. 9–17 = tight space

**Table 5:** Forward stepwise binary logistic regression of prognostic predictors of death within 24 h after admission with active vascular contrast extravasation of the abdomen and pelvis (*n* = 223)

Model	OR (95% CI)	*p*-value	AIC	BIC	AUC (95% CI)
Model 1 (Computed tomographic classes using LPA)^a^			46.663	56.844	0.767 (0.534–1.000)
Slow	Reference				
Moderate	3.644 (0.325, 40.871)	0.294			
Rapid	29.556 (2.442, 357.753)	0.008			
Model 2 (Changes in perimeter of AVCE between AP and PVP; %)^b^			43.135	53.316	0.772 (0.507–1.000)
< 250	Reference				
250–450	12.750 (1.044, 155.675)	0.046			
> 450	34.000 (4.075, 283.669)	0.001			

*AIC* Akaike’s Information Criterion, *BIC* Bayesian Information Criterion

^a^ Includes variables such as diastolic blood pressure, partial thromboplastin time, packed red cell units within 24 h, and computed tomographic classes determined by LPA

^b^ Includes variables such as diastolic blood pressure, partial thromboplastin time, packed red cell units within 24 h, and changes in the area, perimeter, minimum length, and maximum length of AVCE between AP and PVP (%)

### Three classes of AVCEs

Abdominopelvic AVCEs were classified into three groups based on their changes in four size parameters between the AP and PVP phases according to LPA. The slow AVCE showed the smallest degree of change, while the rapid AVCE class exhibited the largest, with significant differences observed among all three classes. Changes in perimeter of AVCEs between AP and PVP had cutoff values at < 250%, 250–450%, and > 450%.

### Characteristics of three AVCE classes (Supplementary Table E3)

**Fig. 3:**
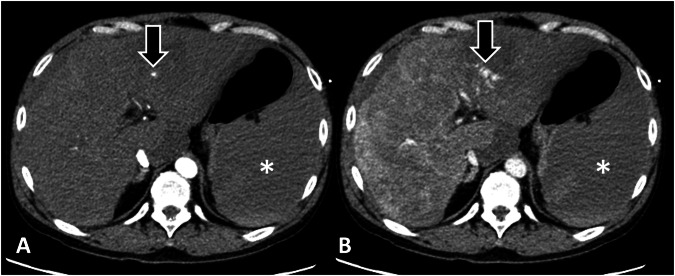
Rapid bleeding and its association with low blood pressures, high packed red cell requirement, and 24-h mortality. Axial CT images in arterial (**A**) and portovenous phases (**B**) of a 62-year-old man show a large ill-defined hepatic mass with a focus of active vascular contrast extravasation (AVCE; arrows) and hemoperitoneum (asterisks). The AVCE is very small in the arterial phase but expands quickly in the portovenous phase, having a change in its perimeter between the two phases of 520%, consistent with rapid bleeding. The patient’s initial blood pressure was 92/54 mmHg with a pulse rate of 70 beats/min. He was treated conservatively with medications and 6 units of red blood cell transfusion and passed away within 24 h of admission

**Fig. 4:**
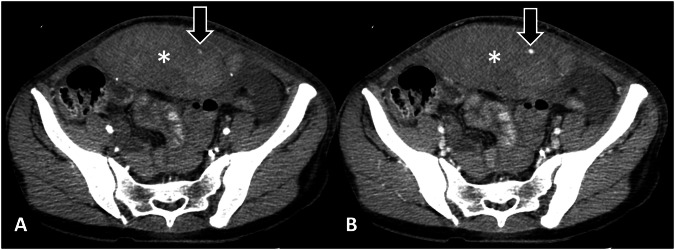
Slow bleeding and its small sizes in the portovenous phase and minimal change between phases. Axial CT images in arterial (**A**) and portovenous phases (**B**) of a 40-year-old woman show a large rectus sheath hematoma (asterisks) with a focus of active vascular contrast extravasation (AVCE; arrows). The AVCE is very small in the arterial phase and changes slightly in the portovenous phase, having a change in its perimeter between the two phases of 22%, consistent with a slow bleeder. The patient’s initial blood pressure was 135/72 mmHg with a pulse rate of 60 beats/min. She was treated successfully with transarterial embolization

Nine factors were unique (distinguishing one AVCE class from the other two): three in the slow class and six in the rapid class. The rapid AVCE (Fig. 3) was associated with the lowest admission systolic and diastolic blood pressures, the highest occurrence of coexistent pseudoaneurysms, and utilized the largest use of red blood cell transfusion within the first 24 h. On CT, rapid AVCEs had the smallest size on AP and the lowest change of mean attenuation between AP and PVP phases. The slow AVCE (Fig. 4) was characterized by the smallest areas on PVP, the shortest maximum length on both PVP and DP, and the smallest changes in minimum diameter between AP and DP phases.

Several factors differentiated between the two AVCE classes, most related to CT size measurements and changes in the size of four size parameters. Rapid AVCEs had a shorter time from CT to treatment but were associated with higher 24-h and in-hospital mortality rates compared to slow AVCEs. Rapid AVCEs showed smaller size parameters on AP but larger measurements on PVP or DP compared to slow AVCEs. Between the slow and moderate AVCEs, the most significant differences were observed in the changes in size parameters between the AP and DP phases. Few clinical parameters were significantly different between rapid vs. slow AVCEs (the former having shorter time from CT to treatment but higher death rates), and moderate vs. rapid AVCEs (the former having higher SBP, DBP, and requiring less RBC transfusion in the first 24 h).

### Identifying the proxy for LPA-derived classes of AVCE

The logistic regression analysis revealed two retained models—one based on the original LPA-derived classification (Model 1) and another using the percentage change in AVCE perimeter between the AP and PVP phases (Model 2)—were retained. A comparison of these models (Table 5) demonstrated superior performance of Model 2, which had a lower AIC (43.135) than Model 1 (AIC = 46.663), indicating a better fit. Similarly, Model 2 had a lower BIC (53.316) than Model 1 (BIC = 56.844), further supporting its improved fit. The AUC of Model 2 (0.772) was also slightly higher than that of Model 1 (0.767). The lower BIC, a more rigorous metric than AIC, underscores that Model 2 provides a more optimal fit. As a result, the percentage change in AVCE perimeter between AP and PVP phases was identified as a practical proxy for the original LPA-derived classification.

### Appearances and alteration in appearances of AVCE in three phases of CT

Most AVCEs (209/223; 93.7%) were detected first in AP CT, and the minority (15/223; 6.7%) had associated pseudoaneurysms or arteriovenous fistulas. 183 AVCEs were confirmed on angiography (*n* = 139) or surgery (*n* = 44), while the rest by multiphase CT. AVCEs progressively enlarged from AP to PVP then DP. Mean CT attenuation and SD, however, slightly dropped from AP to PVP and markedly dropped in DP. The greatest alterations of CT appearances of AVCE were observed between AP and DP for both size and CT attenuation parameters, followed by AP-PVP pair for size, and PVP-DP pair for CT attenuation.

## Discussion

Our investigation identifies three classes of abdominopelvic AVCEs, ranging from slow to rapid-linked to mortality rates, and offers cutoff values using size parameters derived from multiphasic CT for distinguishing these classes with potential clinical relevance.

### Basis for categorizing AVCEs

Previous studies have traditionally categorized AVCEs by anatomical location [3, 5], but only a few [9, 11, 12] have introduced categorization that highlights the varying significance of AVCEs in different groups. Categorizing AVCEs based on mortality data can help raise awareness and inform management practices, as some AVCEs require immediate aggressive management and others may not. Accurately and timely selecting the appropriate AVCEs for transarterial embolization or surgery is crucial.

### Three classes of AVCEs

AVCEs were categorized based on changes in size parameters between the AP and PVP phases of CT, each associated with different mortality risks. The profile of rapid AVCE was most distinctive, characterized by hypotension and the highest RBC transfusion requirements. Rapid AVCEs also showed the shortest time from arrival to imaging and treatment. On multiphase CT, they appeared small on the AP phase—possibly due to low blood pressure—but expanded rapidly on the PVP phase. This highlights the importance of assessing rapid progression between these two phases for identifying rapid AVCEs, in which immediate treatment is vital due to their high mortality rate. These findings underscore the concept of time-conscious bleeding control, in which TAE has an active role [16–18] to reduce blood loss and enhance nonoperative management of hemorrhage [17–20], including those with unstable lower GI bleeding [21, 22].

Patients with slow and moderate AVCEs tended to have a relatively better clinical profile. Time interval parameters suggested less urgency for both imaging and treatment compared to rapid AVCEs. On CT, these two classes were notable for having larger baseline size parameters in the arterial phase than rapid AVCEs, but these size increase was smaller in the portovenous phase. While this may be counterintuitive, it could be explained by the poor hemodynamic status of patients with rapid AVCE, where there is systemic vasoconstriction resulting in contrast medium slowly reaching the bleeding site during the early phase of administration. This phenomenon may be analogous to the hypoperfusion complex, in which the abdominal aorta appears small under such conditions [23, 24].

### Sites, theoretical three spaces, and etiologies of bleeding

In this analysis, the sites, spaces and etiologies of AVCE did not significantly impact mortality, as no major differences were observed across the three AVCE classes. However, in clinical practice, the sites and etiologies of bleeding heavily influence the urgency and type of treatment required. The inability of this analysis to capture the importance of bleeding sites and etiologies could be due to various factors, including the broad inclusion that combines both traumatic and nontraumatic cases and a relatively small sample size. It is well established that bleeding is a primary cause of early death in trauma cases [25], with those with AVCE having 18–32% mortality rates [7, 9, 26, 27]. On the other hand, nontraumatic bleeding-related deaths involving various organs or structures can result from a complex interplay of multiple factors with a wide range of mortality rates between 15 and 75% [11, 28, 29].

### Role of multiphase CT for AVCE

Multiphase CT scans can be valuable for diagnosing conditions like abdominal trauma [30], gastrointestinal hemorrhage [31], and suspected spontaneous abdominal hemorrhages [1], with a key advantage being the ability to detect AVCE more effectively. It provides additional time for AVCE to become visible, distinguishing it from non-bleeding lesions and contained vascular injuries. It also aids in estimating the rate of active bleeding by assessing changes in size among phases and enhances diagnostic confidence for radiologists in both detection and characterization [30]. Our study aligns with classic multiphase CT findings of AVCEs [1, 30, 32]. AVCEs tend to progressively increase in size, exhibit lower CT attenuation, and become less heterogeneous over time, and this trend is consistent across all three AVCE classes.

The study has limitations, including its single-center retrospective design with a relatively small sample size. Identifying patients through the Radiological Information System is not comprehensive and carries a potential for selection bias, as cases without formal CT reports—such as those from outside institutions—may not be captured. Although we attempted to mitigate this by reviewing angiography reports and identifying corresponding CT scans, it remains possible that some patients with active bleeding referred from outside hospitals did not undergo interventional radiology consultation and were therefore missed. The absence of angiographic or surgical confirmation of AVCE may affect study accuracy, even though CT reinterpretation by three radiologists was used as a reference standard; this method is impractical in real-world practice. AVCE is a dynamic process influenced by various factors, making it challenging for any imaging method to provide a comprehensive view. While we acknowledge the differences between nontraumatic and traumatic bleeding—since the latter is rarely associated with anticoagulants or antiaggregant therapy and treatments differ—we chose to analyze all AVCEs regardless of their cause to provide an overall view across a wide spectrum of cases. This approach allows for a broader understanding of our findings, particularly concerning CT imaging. CT analysis relies on one axial image to represent the case, usually the largest size. While 3D volume evaluation is logistically challenging, 2D measurements may be sufficient and feasible in practice. Obtaining the perimeter of AVCE could be somewhat time-consuming in clinical practice, although this functionality is typically available on routine PACS. We did not perform similar measurements on the hematoma in the pre-contrast CT phase, which limits the evaluation of changes in their attenuation in different post-contrast phases. Although we found associations between treatment interval and rapid AVCEs, individual patient treatment should consider multiple clinical factors. Close monitoring and follow-up care are crucial for treatment effectiveness and the prevention of complications. Future studies should focus on validating these findings across larger, multi-institutional cohorts, exploring the role of pre-contrast hematoma measurements, volumetric AVCE measurements, dual-energy CT [33], tailored treatment protocols for different AVCE classes, and investigating specific etiologies of each bleeding to refine management strategies.

In conclusion, active vascular contrast extravasation within the abdomen and pelvis can be categorized into slow, moderate, and rapid classes on multiphasic CT, each associated with distinct 24-h and in-hospital mortality risks. Size-based cutoff values have the potential to guide management decisions and improve clinical outcomes.

## Supplementary information

ELECTRONIC SUPPLEMENTARY MATERIAL

## Abbreviations

AP: Arterial phase
AVCE: Active vascular contrast extravasation
CT: Computed tomography
DP: Delayed phase
HU: Hounsfield unit
ICU: Intensive care unit
LOS: Length of stay
PRC: Packed red cell
PVP: Portovenous phase
TAE: Transarterial embolization
