# Supplementary material for: Rapid, moderate, or slow bleeding? CT analysis of abdominopelvic active vascular contrast extravasation classes and mortality outcomes
Source: Eur Radiol. 2025 May 21;35(11):6669–81. doi: 10.1007/s00330-025-11693-z (PMC12559047; doi:10.1007/s00330-025-11693-z)
Supplement: Supplementary file 1 — ELECTRONIC SUPPLEMENTARY MATERIAL [file 330_2025_11693_MOESM1_ESM.pdf]

Rapid, moderate, or slow bleeding? CT analysis of abdominopelvic active vascular contrast extravasation classes and mortality outcomes

ELECTRONIC SUPPLEMENTARY MATERIAL

Supplementary File 1: Detailed explanation on how the cutoff values of four changes in size parameters were established, using the change in area in AP vs PVP (%) as an example

Classification of change in area between AP and PVP using percentile-based normalization

To stratify the percent change in area between the arterial phase (AP) and portal venous phase (PVP) into three distinct categories (Low, Medium, and High), percentile data derived from three groups identified via latent profile analysis (LPA) were integrated and normalized using a uniform scaling approach.

Percentile distribution by group

Percentile values for each group are presented below:

Group	5th	10th	25th	50th	75th	90th	95th
1	80.03	88.68	167.95	300.81	434.02	663.58	758.25
2	-16.15	-4.36	14.93	58.61	107.95	153.25	197.35
3	748.12	795.95	1109.78	1517.91	1953.41	2738.29	–

Data normalization and classification procedure:

Step 1: Aggregation of percentiles

All percentiles across the three LPA-derived groups were aggregated, resulting in the following dataset:
{80.03, 88.68, 167.95, 300.81, 434.02, 663.58, 758.25, −16.15, −4.36, 14.93, 58.61, 107.95, 153.25, 197.35, 748.12, 795.95, 1109.78, 1517.91, 1953.41, 2738.29}

Step 2: Min-Max normalization
To enable uniform scaling, min-max normalization was applied using the following formula:

$$x_{\text{normalized}} = \frac{x - x_{min}}{x_{max} - x_{min}}$$

Where x is the original value, x_{min}=−16.15, and x_{max}=2738.29. This transformation scales all values to a [0, 1] range.

Step 3: Category assignment

Normalized values were then stratified into three ordinal categories based on equal intervals within the normalized range:

- **Low**: 0.00–0.33
- **Medium**: 0.34–0.66
- **High**: 0.67–1.00

Normalized values and corresponding classifications

Original value	Normalized value	Category
-16.15	0.0000	Low
-4.36	0.0043	Low
14.93	0.0113	Low
58.61	0.0271	Low
80.03	0.0349	Low
88.68	0.0381	Low
107.95	0.0451	Low
153.25	0.0615	Low
167.95	0.0668	Low
197.35	0.0775	Low
300.81	0.1151	Low
434.02	0.1634	Low
663.58	0.2468	Low
748.12	0.2775	Low
758.25	0.2811	Low
795.95	0.2948	Low
1109.78	0.4088	Medium
1517.91	0.5569	Medium
1953.41	0.7151	High
2738.29	1.0000	High

Final categorization thresholds

Based on the normalized values and corresponding clinical distribution, percent change in area between AP and PVP was categorized as follows:

Category	Range of change in area (%)
Low (Slow AVCE)	< 800
Medium (Moderate AVCE)	800–1500
High (Rapid AVCE)	> 1500

Supplementary File 2: Detailed tables 3, 4

Table 3 (detailed): Comparison of patient, treatment, and outcome characteristics among three computed tomographic classes of active vascular contrast extravasation (AVCE) of the abdomen and pelvis (n = 223)

	**All (n = 223)**	**Slow AVCE (n =136)**	**Moderate AVCE (n = 75)**	**Rapid AVCE (n = 12)**	***P*-values**
**Mean age** (years; SD)	59.8 (20.1)	58.3 (19.3)	61.5 (21.6)	65.2 (17.8)	0.344
**Male sex** (n, %)	123 (55.2)	78 (57.4)	41 (54.7)	4 (33.3)	0.275
**Underlying diseases** (n, %)					
Hematologic disease with bleeding tendency	12 (5.4)	8 (5.9)	4 (5.3)	0 (0)	0.687
Chronic kidney disease stage 4-5 or dialysis	35 (15.7)	18 (13.2)	15 (20.0)	2 (16.7)	0.432
Hepatic cirrhosis	22 (9.9)	16 (11.8)	6 (8.0)	0 (0)	0.340
**Medications** (n, %)					
Antiplatelet	37 (16.6)	22 (16.2)	14 (18.7)	1 (8.3)	0.657
Anticoagulant	69 (30.9)	42 (30.9)	23 (30.7)	4 (33.3)	0.983
Herbal	6 (2.7)	2 (1.5)	4 (5.3)	0 (0)	0.212
**Nontraumatic etiologies**	169 (75.8)	99 (72.8)	60 (80.0)	10 (83.3)	0.414
**Vital signs** (mean, SD)					
Systolic blood pressure (mmHg)	123.3 (23.3)	123.8 (24.1)	125.8 (20.7)	**102.9 (20.8)**	0.006
Diastolic blood pressure (mmHg)	72.6 (16.1)	73.3 (15.7)	73.6 (15.6)	**57.3 (17.2)**	0.003
Pulse pressure (mmHg)	50.8 (18.9)	50.5 (19.7)	52.2 (18.4)	45.6 (11.5)	0.513
Pulse rate (beats/min)	95.8 (21.6)	95.4 (21.6)	97.2 (21.5)	92.3 (22.7)	0.703
**Laboratory results** (median, min-max)					
Hemoglobin (g/dL)	8 (3, 38)	7.9 (3, 16)	8.7 (3, 38)	7.4 (4, 10)	0.212
Hematocrit (%)	24 (9, 52)	24.1 (11, 47)	24.7 (9, 52)	21.8 (13, 29)	0.119
Platelet (× 10^3 cell/mm^3)	168 (5, 795)	188 (5, 795)	151 (34, 459)	133 (61, 356)	0.512
Prothrombin time (sec) (n = 199)	14.7 (10.2, 64.2)	14.3 (10.2, 43.9)	15.0 (10.3, 64.2)	15.5 (13.4, 28.8)	0.120
Partial thromboplastin time (sec) (n = 199)	27.5 (12, 144)	28.2 (18, 67)	26.6 (12, 144)	28.4 (23, 121)	0.337
International normalized ratio (n = 145)	1.4 (1, 7)	1.4 (1, 7)	1.4 (1, 7)	1.5 (1, 3)	0.742
Base excess (n = 71)	-5.9 (-26, 12)	-5.3 (-26, 12)	-7.4 (-13, 4)	-9.6 (-25, 0)	0.439
Lactate (mg/dL) (n = 125)	4.3 (1, 24)	4.3 (1, 24)	3.7 (1, 18)	6.3 (2, 19)	0.307
**Packed red cells**					
Use (n, %)	183 (82.1)	106 (77.9)	67 (89.3)	10 (83.3)	0.118
Units in 24 hours (median, min-max)	3 (0, 21)	3 (0, 17)	3 (0, 17)	**6 (0, 21)**	0.005

**Time intervals** (hours; median, min-max)					
From CT to angiography	5.4 (0.4, 148.3)	6.2 (0.4, 123.7)	4.8 (0.4, 148.3)	2.7 (1.1, 8.4)	0.096
From CT to surgery or other procedures	16.1 (0.4, 312.6)	18.4 (0.9, 201.7)	8.4 (0.4, 312.6)	3.5 (2.4, 17.5)	0.207
From CT to any treatment	5.3 (0.4, 201.7)	*6.1 (0.4, 201.7)*	4.9 (0.4, 165.5)	*2.8 (1.1, 8.4)*	0.046
**Angioembolization** (n, %) (n=138)	123 (89.1)	74 (90.2)	44 (88.0)	5 (83.3)	0.492
**Surgery** (n, %)	44 (19.9)	26 (19.3)	14 (18.7)	4 (36.4)	0.475
**Pharmacological adjuncts** (n, %)	83 (37.6)	46 (34.1)	30 (40.0)	7 (63.6)	0.201
**Other procedures** (n, %)	29 (13.1)	16 (11.9)	12 (16.0)	1 (9.1)	0.603
**None of above treatments** (n, %)	25 (11.3)	15 (11.1)	8 (10.7)	2 (18.2)	0.758
**Treatment combination**					0.603
Angioembolization alone	56 (25.1)	39 (28.7)	17 (22.7)	0	
Angioembolization with other treatment	67 (30.0)	35 (25.7)	27 (36.0)	5 (41.7)	
Other treatment not involving angioembolization	60 (26.9)	39 (28.7)	17 (22.7)	4 (33.3)	
None of above treatments	40 (17.9)	23 (16.9)	14 (18.7)	3 (25.0)	
**ICU admission** (n, %)	129 (57.8)	74 (54.4)	48 (64.0)	7 (58.3)	0.402
**Length of stay** (days; median, min-max)					
Non-ICU ward	11 (0, 340)	10.5 (0, 340)	11 (0, 179)	32 (0, 101)	0.460
ICU	2 (0, 170)	1 (0, 113)	4 (0, 170)	2.5 (0, 28)	0.180
From CT to discharge	12 (0, 356)	12 (1, 356)	13 (0, 180)	12.5 (0, 115)	0.700
Total	17 (0, 355)	15.5 (0, 355)	18 (1, 179)	32 (1, 129)	0.142
**Follow-up CT**	104 (46.6)	64 (47.1)	34 (45.3)	6 (50.0)	0.944
**Rebleeding** (n, %)	24 (10.9)	15 (11.2)	7 (9.3)	2 (16.7)	0.736
**Discharge status** (n, %)					0.011
Improved	140 (62.8)	90 (66.2)	45 (60.0)	5 (41.7)	
Dead	55 (24.7)	*25 (18.4)*	23 (30.7)	*7 (58.3)*	
Transferred	28 (12.6)	21 (15.4)	7 (9.3)	0 (0)	
**Death** (n, %)					
within 24 hours of index CT	5 (2.2)	*1 (0.7)*	2 (2.7)	*2 (16.7)*	0.002
>24 hours to 7 days	13 (5.8)	7 (5.1)	4 (5.3)	2 (16.7)	0.257
>7 days	37 (16.6)	17 (12.5)	17 (22.7)	3 (25.0)	0.119
Within same admission (n, %)	55 (24.7)	*25 (18.4)*	23 (30.7)	*7 (58.3)*	0.003

p-values of < 0.05 are marked with underline.

The light gray boxes (values in bold) indicate values that exhibit a significant difference from the other two AVCE classes in a pairwise comparison.

The gray boxes (values in *italics*) indicate same-row pairs that exhibit a significant difference in a pairwise comparison.

Table 4 (detailed): Comparison of computed tomography characteristics among three classes of active vascular contrast extravasation (AVCE) of the abdomen and pelvis (n = 223)

	All (n = 223)	Slow AVCE (n =136)	Moderate AVCE (n = 75)	Rapid AVCE (n = 12)	*p*-value
**Time from event to CT** (hours; median, min-max)	5.2 (0.1, 685.7)	4.9 (0.1, 685.7)	6.4 (1.0, 352.4)	6.8 (1.9, 30.1)	0.398
**Site of AVCE*** (n, %)					0.852
Gastrointestinal	51 (22.9)	26 (19.1)	22 (29.3)	3 (25.0)	
Peritoneum	30 (13.5)	20 (14.7)	9 (12.0)	1 (8.3)	
Mesentery	5 (2.2)	2 (1.5)	2 (2.7)	1 (8.3)	
Post surgical spaces	6 (2.7)	4 (2.9)	2 (2.7)	0 (0)	
Extraperitoneum	11 (4.9)	5 (3.7)	5 (6.7)	1 (8.3)	
Retroperitoneum	40 (17.9)	23 (16.9)	14 (18.7)	3 (25.0)	
Spleen	2 (0.9)	2 (1.5)	0 (0)	0 (0)	
Subcutaneous tissues	7 (3.1)	6 (4.4)	1 (1.3)	0 (0)	
Adrenal glands	2 (0.9)	2 (1.5)	0 (0)	0 (0)	
Muscles	31 (13.9)	19 (14.0)	11 (14.7)	1 (8.3)	
Non-fat-containing	1 (0.4)	0 (0)	1 (1.3)	0 (0)	
tumors	5 (2.2)	3 (2.2)	1 (1.3)	1 (8.3)	
Kidneys	14 (6.3)	9 (6.6)	4 (5.3)	1 (8.3)	
Liver	1 (0.4)	1 (0.7)	0 (0)	0 (0)	
Ovary	1 (0.4)	0 (0)	1 (1.3)	0 (0)	
Pancreas	15 (6.7)	13 (9.6)	2 (2.7)	0 (0)	
Rectus sheath	1 (0.4)	1 (0.7)	0 (0)	0 (0)	
Uterus					
**AVCE grouped by ease of spread*** (n, %)					0.673
Free	92 (41.3)	52 (38.2)	35 (46.7)	5 (41.7)	
Loose	60 (26.9)	36 (26.5)	20 (26.7)	4 (33.3)	
Tight	71 (31.8)	48 (35.3)	20 (26.7)	3 (25.0)	
**Number of AVCE in organ of interest** (median, min-max)	1 (1, 10)	1 (1, 10)	1 (1, 8)	1 (1, 4)	0.161
**AVCE first shown in arterial phase** (n, %)	209 (93.7)	129 (94.9)	69 (92.0)	11 (91.7)	0.684
**Coexistent pseudoaneurysm** (n, %)	15 (6.7)	6 (4.4)	5 (6.7)	**4 (33.3)**	<0.001
**Angiographically positive AVCE** (n, %)	138 (61.9)	82 (60.3)	50 (66.7)	6 (50.0)	0.478
**Surgically confirmed AVCE** (n, %)	44 (19.9)	26 (19.3)	14 (18.7)	4 (36.4)	0.372
**Area of AVCE** (mm^2; median, min-max)					
AP (n = 209)	47.9 (1.3, 571.2)	54.3 (1.3, 571.2)	44.2 (6.3, 486.9)	**18.4 (4.2, 102.8)**	0.007
PVP (n = 219)	120.6 (2.5, 962.6)	**102.7 (2.5, 882.2)**	141.3 (5.7, 962.6)	196.3 (82.4, 500.1)	0.001
DP (n = 145)	208.6 (13.2, 2792.7)	*155.5 (13.2, 1673.1)*	253.9 (25.4, 2792.7)	*298.9 (208.6, 1788.6)*	0.035
∆ between AP and PVP (%)	171.2 (-40.2, 3500)	**58.6 (-40.2, 223.7)**	**300.8 (27.6, 841.4)**	**1517.9 (748.1, 3005)**	<0.001
∆ between AP and DP (%)	313.8 (-77.4, 7825.7)	**183.0 (-77.4, 2064.1)**	550.7 (-36.0, 3364.1)	4376.4 (929.5, 7825.7)	<0.001
∆ between PVP and DP (%)	73.0 (-83.1, 1181.1)	72.1 (-83.1, 1181.1)	61.3 (-68.7, 459.7)	84.6 (-14.2, 278.3)	0.814
**Perimeter of AVCE** (mm; median, min-max)					

AP (n = 209)	39.6 (10.0, 192.1)	*43.2 (10.0, 192.1)*	35.6 (10.9, 168.7)	*21.1 (10.2, 73.2)*	0.014
PVP (n = 219)	60.2 (11.1, 312.9)	**52.6 (11.2, 312.9)**	65.5 (11.1, 278.8)	106.7 (40.9, 156.8)	<0.001
DP (n = 145)	80.2 (15.5, 352.6)	*71.2 (15.5, 352.6)*	91.7 (25.0, 337.6)	*107.9 (73.3, 305.9)*	0.007
∆ between AP and PVP (%)	40.2 (-89.1, 770.6)	**17.0 (-36.2, 91.8)**	**111.0 (-89.1, 299.2)**	**477.1 (253.0, 770.6)**	<0.001
∆ between AP and DP (%)	93.1 (-70.5, 1353.1)	**57.3 (-70.5, 395.0)**	175.4 (-34.4, 491.6)	836.0 (230.7, 1353.1)	<0.001
∆ between PVP and DP (%)	28.5 (-68.4, 272.5)	29.2 (-68.4, 272.5)	27.5 (-63.4, 217.9)	50.6 (-40.0, 134.3)	0.866
**Minimum length of AVCE** (mm; median, min-max)					
AP (n = 209)	6.6 (0.7, 39.9)	*7.3 (0.7, 39.9)*	5.7 (2.1, 22.0)	*3.8 (2.0, 10.1)*	0.002
PVP (n = 219)	10.3 (1.1, 45.2)	*9.5 (1.1, 45.2)*	12.5 (2.2, 43.5)	*13.9 (7.8, 29.9)*	0.011
DP (n = 145)	13.7 (3.8, 61.9)	13.2 (3.8, 46.7)	15.5 (3.9, 58.4)	15.2 (12.2, 61.9)	0.217
∆ between AP and PVP (%)	43.9 (-48.6, 484.1)	**22.1 (-48.6, 100.7)**	**113.5 (-16.5, 416.8)**	**342.1 (281.8, 484.1)**	<0.001
∆ between AP and DP (%)	101.0 (-63.8, 1485.4)	**65.9 (-63.8, 383.1)**	195.7 (-20.6, 662.9)	640.8 (267.0, 1485.4)	0.002
∆ between PVP and DP (%)	30.2 (-61.9, 378.2)	32.1 (-61.9, 378.2)	26.6 (-45.1, 265.7)	58.2 (-11.8, 315.3)	0.757
**Maximum length of AVCE** (mm; median, min-max)					
AP (n = 209)	13.3 (3.2, 58.0)	*14.2 (3.2, 48.3)*	12.6 (3.5, 58.0)	*7.4 (3.2, 26.2)*	0.037
PVP (n=219)	20.4 (3.5, 81.4)	**18.4 (3.5, 81.4)**	24.1 (3.8, 70.8)	35.8 (12.0, 53.8)	<0.001
DP (n = 145)	28.4 (5.0, 107.5)	**24.2 (5.0, 107.5)**	32.2 (7.8, 87.5)	38.6 (22.3, 91.7)	0.002
∆ between AP and PVP (%)	34.5 (-36.1, 786.4)	**19.7 (-36.1, 108.2)**	**114.3 (-18.8, 317.1)**	**440.8 (157.8, 786.4)**	<0.001
∆ between AP and DP (%)	88.5 (-60.3, 1288.0)	*59.5 (-60.3, 427.2)*	174.5 (-35.8, 423.4)	*740.5 (203.5, 1288.0)*	<0.001
∆ between PVP and DP (%)	23.2 (-71.3, 316.8)	20.5 (-62.7, 316.8)	31.2 (-71.3, 186.6)	33.3 (-44.0, 163.8)	0.876
**Mean attenuation of AVCE** (HU; median, min-max)					
AP (HU; n = 209)	128.3 (61.0, 480.4)	125.2 (61.0, 347.7)	131.4 (62.4, 329.0)	142.0 (90.6, 480.4)	0.735
PVP (HU; n = 219)	125.3 (52.8, 441.5)	123.0 (55.3, 313.6)	132.0 (52.8, 441.5)	103.4 (65.6, 204.3)	0.075
DP (HU; n = 145)	96.2 (53.2, 177.2)	92.0 (55.0, 167.2)	103.0 (53.2, 177.2)	86.8 (63.2, 143.0)	0.144
∆ between AP and PVP (%)	-7.5 (-80, 364.3)	-7.7 (-80, 280.9)	-2.4 (-50.9, 364.3)	**-21.8 (-59.4, 12.8)**	0.011
∆ between AP and DP (%)	-28.1 (-77.7, 79.8)	-29.1 (-70.9, 45.4)	-26.7 (-73.1, 79.8)	-36.6 (-77.7, -4.7)	0.303
∆ between PVP and DP (%)	-21.7 (-63.9, 87.4)	-21.7 (-63.9, 87.4)	-23.0 (-62.8, 40.0)	-18.4 (-47.5, 14.4)	0.639
**Standard deviation of attenuation of AVCE** (HU; median, min-max)					
AP (HU; n = 189)	54.9 (19.5, 182.1)	54.4 (19.5, 145.5)	55.1 (21.0, 182.1)	59.6 (48.6, 102.5)	0.479
PVP (HU; n = 215)	50.2 (16.1, 252.6)	48.0 (17.1, 120.4)	54.9 (16.1, 252.6)	49.1 (32.8, 61.7)	0.232
DP (HU; n = 145)	35.0 (13.9, 87.4)	*33.6 (13.9, 71.8)*	*41.2 (22.0, 87.4)*	35.0 (25.7, 40.8)	0.037
∆ between AP and PVP (%)	-7.9 (-77.3, 313.9)	*-11.9 (-77.3, 196.0)*	*12.2 (-51.9, 313.9)*	-24.6 (-43.8, -6.9)	0.001
∆ between AP and DP (%)	-31.8 (-76.8, 57.3)	*-39.7 (-76.8, 57.3)*	*-16.7 (-69.2, 47.1)*	NA	0.002
∆ between PVP and DP (%)	-30.7 (-74.2, 24.9)	-30.1 (-70.8, 24.9)	-25.9 (-74.2, 18.9)	-36.6 (-49.2, -4.9)	0.632

AP arterial phase, *AVCE* active vascular contrast extravasation, *DP* delayed phase, *NA* not applicable, *PVP* portovenous phase, *HU* Hounsfield unit, *SD* standard deviation

p-values of < 0.05 are marked with underline.

*No. 1-4 = free space; No. 5-8 = loose space; No. 9-17 = tight space

The light gray boxes (values in bold) indicate values that exhibit a significant difference from the other two AVCE classes in a pairwise comparison.

The gray boxes (values in *italics*) indicate same-row pairs that exhibit a significant difference in a pairwise comparison.

SUPPLEMENTARY TABLES

Supplementary Table E1: Definitions and examples of grouping of active vascular contrast extravasation based on perceived ease of spread

	**Definitions**	**Examples**
**Free space**	Easy spread of leaked blood products	Gastrointestinal tract, peritoneal cavity
**Loose space**	Intermediate ability of leaked blood products to spread	Retroperitoneum, extraperitoneum, subcapsular spaces of solid organs, subcutaneous tissue, fat-containing solid tumor, spleen
**Tight space**	Limited ability of leaked blood products to spread	Solid organs, muscles, non-fat-containing solid tumors

Supplementary Table E2: Model- fit statistics of latent profile analysis for correct number of latent classes in Method #6

Number of mixture components	Log-likelihood	BIC	ICL	BLRT	*P*-value
1 vs. 2	-4612.358	-9365.301	-9393.424	197.947	0.001
2 vs. 3	-4576.630	-9326.289	**-9374.049**	71.456	0.001
3 vs. 4	-4560.376	**-9326.225**	-9389.148	32.507	0.002
4 vs. 5	-4560.406	-9358.727	-9427.13	-0.059	0.542

The p-value associated with the BLRT.

The number of classes that yielded the best BIC or ICL value appears in bold.

BIC Bayesian Information Criterion, *BLRT* bootstrap likelihood ratio test, *ICL* integrated complete-data likelihood

Supplementary Table E3: Summary of unique and differentiating factors of three computed tomographic classes of active vascular contrast extravasation (AVCE) of the abdomen and pelvis*

	Slow AVCE	Medium AVCE	Rapid AVCE
**Unique[1] characteristics**	*Smallest area on PVP*<br>*Smallest max length on PVP and DP*<br>*Smallest Δ min length in AP-DP pair*	-	Lowest sBP<br>Lowest dBP<br>*Coexistent pseudoaneurysm*<br>*Smallest area on AP*<br>*Lowest Δ mean HU in AP-PVP pair*<br>Largest number of RBC units in 24 hours
**Differentiating[2] characteristics**	**Vs. Medium AVCE**<br>*Smaller area in PVP*<br>*Smaller perimeter in PVP*<br>*Smaller max length in PVP*<br>*Smaller max length in DP*<br>*Lower SD HU in DP*<br>*Smaller Δ area in AP-DP pair*<br>*Smaller Δ perimeter in AP-DP pair*<br>*Smaller Δ min length in AP-DP pair*<br>*Smaller Δ SD HU in AP-DP pair*<br>*Larger Δ SD HU in AP-PVP pair*	**Vs. Rapid AVCE**<br>Higher sBP<br>Higher dBP<br>Lesser RBC units in 24 hrs<br>*Larger area in AP*<br>*Smaller Δ mean HU in AP-PVP pair*	**Vs. Slow AVCE**<br>*Smaller area, perimeter, min length, max length in AP*<br>*Larger area, perimeter, min length in PVP*<br>*Larger area, perimeter in DP*<br>*Larger Δ max length in AP-DP pair*<br>Shorter time from CT to Rx<br>More deaths in 24 hours after index CT and same admission

AP arterial phase, *CT* computed tomography, *dBP* diastolic blood pressure, *DP* delayed phase, *HU* Hounsfield units, *PVP* portovenous phase, *sBP* systolic blood pressure, *SD* standard deviation

*Factors identified on CT are presented in *italics*

[1] Differences among active vascular contrast extravasation in that class from other classes

[2] Differences between pairs of active vascular contrast extravasation in three classes
